# Non-Carbon 2D Materials-Based Field-Effect Transistor Biosensors: Recent Advances, Challenges, and Future Perspectives

**DOI:** 10.3390/s20174811

**Published:** 2020-08-26

**Authors:** Mohammed Sedki, Ying Chen, Ashok Mulchandani

**Affiliations:** 1 Department of Materials Science and Engineering, University of California, Riverside, CA 92521, USA; mabue002@ucr.edu; 2 Department of Chemical and Environmental Engineering, University of California, Riverside, CA 92521, USA; ychen751@ucr.edu

**Keywords:** 2D-materials, field-effect transistor, transition metal dichalcogenides, black phosphorus, phosphorene, hexagonal boron nitride, transition metal oxides, biosensors

## Abstract

In recent years, field-effect transistors (FETs) have been very promising for biosensor applications due to their high sensitivity, real-time applicability, scalability, and prospect of integrating measurement system on a chip. Non-carbon 2D materials, such as transition metal dichalcogenides (TMDCs), hexagonal boron nitride (h-BN), black phosphorus (BP), and metal oxides, are a group of new materials that have a huge potential in FET biosensor applications. In this work, we review the recent advances and remarkable studies of non-carbon 2D materials, in terms of their structures, preparations, properties and FET biosensor applications. We will also discuss the challenges facing non-carbon 2D materials-FET biosensors and their future perspectives.

## 1. Introduction

Field-effect transistors (FETs) are highly promising for biosensor applications due to their high sensitivity, real-time applicability, scalability, and prospect of integrating measurement system on a chip. A conventional FET system is composed of two electrodes, source and drain, connected by a semiconducting channel material. The FET sensor responds based on conductance change of the semiconductor channel material due to a gating effect of the captured analyte molecules. This gating effect modulates the electrical characteristics of the FET, such as source-to-drain current. This change in FET characteristics is transduced as a detectable signal change [1]. Bulk materials, such as gas-sensitive metal oxides and polymer membranes, were used as the first channel materials in FET chemical sensors. However, the unfavored electronic properties and limited interaction between target molecules and bulky materials limited their use, especially as they sometimes require specific operating conditions, such as high temperature for gas sensing [2,3,4].

One-dimensional (1D) semiconducting nanomaterials, such as carbon nanotubes (CNTs), conducting polymer nanowires (CPNWs) and silicon nanowires (SiNWs) have shown great success as channel materials in FET sensors. The high sensitivity of the 1D-FET sensors is attributed to their high surface area and high switching characteristics (current on/off ratio) of CNTs, CPNWs and SiNWs [5,6,7]. Despite the application of SiNWs-FET in many biosensor applications, it is challenging to scale up or commercialize them due to the low carrier mobility of SiNWs and the high device-to-device variation [8]. Even with the superior physicochemical properties of CNTs, such as excellent thermal and chemical stability [9], exceptional conductivity [10,11], and feasibility to easily immobilize bioprobes, their applications to FET sensors are limited. This can be explained by the difficulty to obtain pure semiconducting (s) or metallic (m) CNTs instead of producing a mixture of them, which destroys the electrical performance and increases the device-to-device variation [8,12].

Two-dimensional (2D) semiconducting nanomaterials, on the other hand, provide more conformal and stronger contact with electrodes. They are also easier to implement because of their relatively larger lateral sizes, which enables better control of the FET channel structure. Moreover, 2D nanosheets can be prepared in the desired shape, size, and thickness, and can be precisely transferred to the designated area of the sensor substrate [1]. Graphene, the most widely used 2D material, has a very high surface area of approximately 2630 m^2^/g [13] and an exceptional mechanical strength [14] (130 GPa tensile strength and 1000 GPa modulus). Moreover, graphene has an ultra-high ideal charge carrier mobility of 200,000 cm^2^ V^−1^ s^−1^, entitling it to have excellent electrical properties of fast electron transfer [15]. This increased attention to graphene as an excellent material for FET applications [16,17,18]. However, the lack of intrinsic band gap in graphene, resulting in a small current on/off ratio in its FETs, limits its sensitivity and applicability in FET sensors [8].

Acknowledging all this, other families of non-carbon 2D materials have been inaugurated and are growing rapidly at present. Analogous with graphene, monolayer and few-layer transition metal dichalcogenides (TMDCs) (e.g., MoS_2_, WS_2_, MoTe_2_, MoSe_2_, WSe_2_), hexagonal boron nitride (h-BN), black phosphorus (BP), transition metal oxides (LaMnO_3_, LaVO_3_), transition metal chalcogenides (NbSe_3_, TaSe_3_), and layered complex oxides have been reported [19,20,21,22,23]. Moreover, other 2D materials, such as silicene and germanene, have been introduced and studied [24]. The huge pool of non-carbon 2D materials covers a large number of materials with a huge variation in properties, from insulators to conductors. More details on the growing library of the 2D materials can be found in these references [25,26]. Therefore, researchers have taken those materials into many applications, including FET-based biosensors. In this article, we review the recent non-carbon 2D materials, in terms of their structures, preparations, properties and their FET biosensors. We will also discuss the challenges facing non-carbon 2D materials-FET biosensors and their future perspectives.

## 2. FET Platform: General Features

A biosensor is an analytical device consisting of a transducer and biological receptor as basic components that convert a biochemical response into an electronic signal. FET devices are popularly used in the electrical biosensing field due to their unique function for weak-signal and high impedance applications [27,28]. In FET-based biosensors, the gate terminal and/or the dielectric layer are modified with specific bioreceptors (antibodies, oligonucleotides, peptides, receptors, cells, enzymes, aptamers, etc.) to capture desired bio/chemical molecules [28,29,30]. When target bio/chemical molecules bind with bioreceptors, surface charges result in modulation of the electrical characteristics of FET devices.

**Figure 1:**
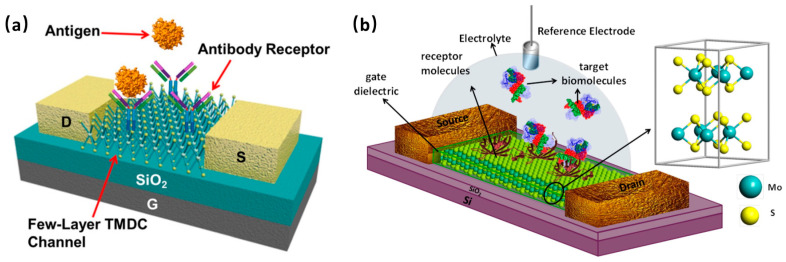
(**a**) Schematic illustration of the back-gated field-effect transistors (FET) biosensor with a few-layer transition metal dichalcogenide (TMDC) sensing channel [33]. Reprinted with permission from ref [33]. Copyright 2015 American Vacuum Society. (**b**) Schematic diagram of liquid-gating MoS_2_-based FET biosensor [34]. Reprinted with permission from ref [34]. Copyright 2014 American Chemical Society. MoS_2_ can be exchanged by other non-carbon 2D materials such as TMDCs, black phosphorus (BP) or metal oxides while antibodies can be swapped with other bioreceptors such as oligonucleotide probes, receptors, enzymes, cells or aptamers.

The most facile and common way to build a 2D-FET platform is to use a wafer-based back-gated configuration as shown in Figure 1a. In this configuration, the deposition of electrodes (source and drain) is needed, while the bulk wafer could directly act as a back gate [31]. Two-dimensional materials could be grown or transferred via typical methods on a wafer of dielectric material (such as SiO_2_) deposited on conducting substrate (such as Si) followed by deposition of metal source and drain electrodes via microfabrication process. Another widely used configuration in exploring 2D-FET biosensors is liquid-ion gating. Different from the back-gated configuration, in which bulk wafer functions as the gate, in this arrangement (as shown in Figure 1b), the ionic liquid is the gate. Hence, a double-layer is generated at the liquid-channel interface that is also the dielectric for the screening of the field [32].

To detect biomolecules with specificity via 2D-FET platforms, the semiconductor channel layer has to be functionalized with biorecognition molecules specific for the target using compatible physicochemical methods [35,36]. Biological interactions of enzyme–substrate, antibody–antigen, complementary nucleic acid strands, etc., are utilized in the FET biosensors to detect target biomolecule with exquisite specificity [37]. When target molecules interact with the bioreceptor molecules, the biological interactions may cause changes in the surrounding chemical environment or material chemical structures and compositions [38]. These changes make an immediate impact to the accumulated charge carriers at the surface of the gate to transduce the biochemical interaction into an electrical signal as a measurable source-drain current. This is the principle of typical FET biosensors.

## 3. Non-Carbon 2D Materials

Table 1 provides an overview of the different non-carbon 2D materials, their electronic/device properties, and their FET-based biosensors.

### 3.1. 2D TMDCs Materials

#### 3.1.1. Structure, Preparation and Properties

TMDCs are a group of layered materials with the general formula MX_2_, where M is a transition metal from groups IV, V, or VI (Ti, Zr, Hf; V, Nb, Ta; Ct, Mo, or W) and X is a chalcogen atom (S, Se, or Te). Each layer of TMDC is composed of three planes: chalcogen, transition metal, and chalcogen. TMDCs exist in different coordination, where each transition metal atom is coordinated to six chalcogen atoms either in an octahedron or triangular prism [19,48]. The type of metal coordination preferred by these materials is highly influenced by the nature of the bond formed between the metal and chalcogen atoms. Essentially, octahedral coordination is favored by group IV transition metals, as they form strong ionic compounds, which have Coulomb repulsive forces between layers. On the other hand, group VI transition elements form more covalent bonds and coordinate in triangular prism [49,50]. Yet, group V transition elements can stabilize in octahedron and triangular prism structures due to their moderate ionicity [48].

TMDCs can be found in one of three stackings or polytypes; 1T-type stacking that dominates in bulk crystals in octahedral coordination, 2H- and 3R-type which are found with the triangular prismatic coordination. Moreover, the stable phase of MX_2_ material at ambient pressure and temperature is the 2H phase with six atoms per unit cell; two metal atoms and four chalcogenides, though 1T phase can be prepared by electron beam irradiation or Li-intercalation [51,52]. It is also worth mentioning that TMDCs may undergo dimerization of metal atoms, which induces the movement of chalcogen atoms to an out-of-plane direction and results in the distortion of the 1T phase to the 1T’ structure. This can be described also by means of symmetry transformation from 3- to 2-fold, with a change in the space group from (P3m1) in 1T to (P2_1_/m) in 1T’ [53].

TMDCs can be prepared in different methods that can be divided into two main approaches; top-down, in which the bulky crystals are exfoliated into mono-/few layered-TMDCs, and bottom-up, in which a thin layer of the material is built from atoms of precursors [54]. Based on these two approaches, there are several techniques/methods introduced to prepare high-quality thin layers TMDCs, including liquid phase exfoliation [55], mechanical exfoliation [56], chemical exfoliation [57,58], electrochemical deposition, and chemical vapor deposition (CVD) [25,59]. Due to its ability to prepare high quality large TMDCs layers, with a controllable number of layers and domain size, CVD is very promising among all aforementioned methods. There are many details and ongoing progress in these methods, especially in CVD-based methods, and for this, we recommend reading the work of You et al. [60] and Zhang et al. [61].

TMDC materials exhibit a wide range of electrical properties, based on the type of phase and the number of d electrons, such as metallic (e.g., NbS_2_, VSe_2_) [62,63], semi metallics (e.g., WTe_2_, TiSe_2_) [64,65], semiconductors (e.g., MoS_2_, MoSe_2_, WS_2_, WSe_2_) [66,67,68], and insulators (e.g., HfS_2_) [69]. The first semiconducting to attract attention among the TMDCs is MoS_2_, which shows a high on/off current ratio [70] that entitles it to be a good candidate for field-effect transistor applications [71]. Except for a few cases of GaSe and ReS_2_, most of the TMDCs such as MoS_2_ (1.8 eV), WS_2_ (2.1 eV) and WSe_2_ (1.7 eV) exhibit an indirect band gap with smaller energies in bulk form and a higher direct band gap in monolayer [72,73]. Nevertheless, most TMDCs, such as MoS_2_ and WSe_2_, are free of dangling bonds and hence make more ideal Schottky junctions than bulk semiconductors. This in turn inhibits charge transfer at the interface with bulk metals by producing Fermi energy pinning and recombination centers [74]. However, some of them exhibit high mobility, depending on metal contacts, selection of the appropriate substrate, grain boundaries, etc. MoS_2_ provides a mobility of 33–151 cm^2^ V^−1^ s^−1^ on BN/Si substrate at room temperature and 700 cm^2^ V^−1^ s^−1^ on SiO_2_/Si substrate with scandium contact [75,76]. In addition, the chemically prepared 1T MoS_2_ phase is 10^7^ times more conductive than its semiconducting 2H phase. On the other hand, the dichalcogenides of Ti, Ni, V, Cr, Zn, Nb essentially exhibit metallic behavior [77]. For FET, the channel material is required to be a semiconductor, as discussed in Section 2, so the semiconducting TMDCs are good candidates for FET. On the other hand, the semimetallic and metallic TMDCs are not good channel materials for FETs, and they are better candidates for electrochemical sensors.

Superiority of TMDCs over graphene. Graphene has interesting properties that have attracted huge attention since its discovery in 2004. However, graphene does not have an intrinsic band gap, which limits its uses in the electronics industry. On the other hand, TMDCs show a tunable band gap that controls the current flow with a high on/off ratio and hence they serve as good materials for transistor applications. For example, MoS_2_ shows direct bandgap (≈1.8 eV), large optical absorption in monolayer (≈10^7^ m^−1^ in the visible range), and high current on/off ratio of ≈10^7^–10^8^. Accordingly, it has been applied extensively in electronics and optoelectronics [78,79].

#### 3.1.2. TMDCs-FET Biosensors

FET-based sensors are electrical systems that depend on the changes in the electrical conductivity of the semiconducting channel materials upon stimulation by target molecules. Therefore, the semiconducting TMDCs, such as MoS_2_, MoSe_2_, WS_2_, and WSe_2_, are the target materials among all the other TMDCs for FET sensors. Semiconducting TMDC-based FET sensors, especially MoS_2_-FETs have several advantages over other materials, such as low leakage current, low power consumption, and high current on/off ratio enabling high sensitivity [80,81]. Moreover, due to their excellent abovementioned electronic properties, and mechanical flexibility, as well as their ultrathin structure, MoS_2_-FET sensors are promising for the economic and low energy portable and wearable electronics [62,82]. We will discuss some of the reported TMDC-FET biosensors for the detection of different targets, such as DNA, glucose, protein, and antibiotics.

**Figure 2:**
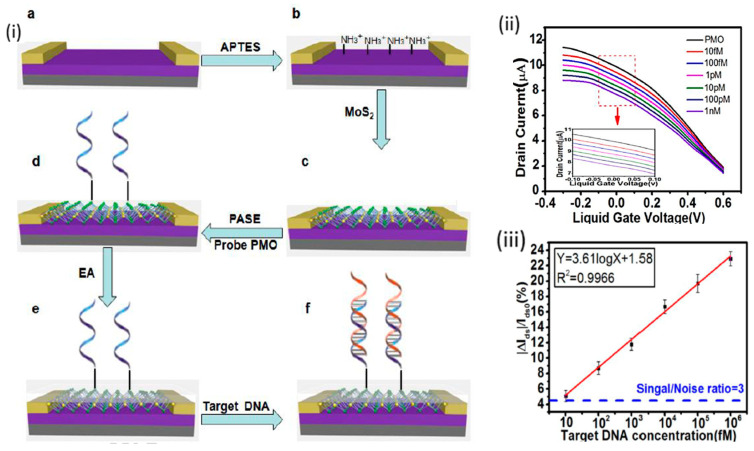
(**i**) A Schematic of the preparation of the MoS_2_ FET biosensor for the detection of DNA; SiO_2_/Si substrate with metal contacts (**a**), APTES functionalization of the substrate (**b**), MoS_2_ loading (**c**), functionalization of PMO on MoS_2_ surface using PASE linker (**d**) exposed surface passivation/blocking using EA (**e**), and target DNA capturing using the sensor (**f**). (**ii**) FET transfer characteristics of the complementary DNA-hybridized phosphorodiamidate morpholino oligos (PMO)-functionalized MoS_2_ FET device, at a series of concentrations. (**iii**) Calibration/working curve of the MoS_2_ FET at different concentrations of DNA. Reprinted from ref [83], Copyright (2018), with permission from Elsevier.

Mei et al. [83] reported the detection of DNA via hybridization with phosphorodiamidate morpholino oligos (PMO), as an ultrasensitive label-free MoS_2_-FET biosensor. As shown in Figure 2i, sensor fabrication was conducted by drawing gold electrodes using photolithography and e-beam evaporation, followed by treatment with 3-Aminopropyltriethoxysilane (APTES) to cover the SiO_2_/Si surface with positive charges. The negatively charged MoS_2_ nanosheets were drop-casted to the positively charged channel surface and bound to it via electrostatic attraction. Then, the MoS_2_ surface was modified with the DNA analogue, PMO, using 1-Pyrenebutanoic acid succinimidyl ester (PASE). The prepared PMO-MoS_2_-FET biosensor showed a low limit of detection (LOD) of DNA of 6 fM, which is lower than other formerly reported DNA–DNA hybridization-based MoS_2_ FET DNA biosensor. This can be attributed to the high sensitivity of the MoS_2_-FET sensor and the successful and selective hybridization with PMO. Moreover, this sensor system showed applicability in the detection of DNA in serum. The signal change was recorded from the change in device current due to stimulation by the target DNA, as shown in the FET characteristic curve in Figure 2ii, and the calibration curve in Figure 2iii. Nevertheless, this system still needs more work to control the reproducibility, and the authors of this work plan to conduct it in the future. Other MoS_2_-FET sensors were introduced for DNA detection, such as Lee et al.’s work in which a LOD of 10 fM was achieved [84]. Furthermore, an earlier work reported by Loan et al. used the heterostructure of MoS_2_/graphene for DNA hybridization detection on FET biosensor, and they were able to achieve a very low LOD in the attomolar range [85].

Majd et al. [39] developed a MoS_2_-FET biosensor for the label-free detection of a breast cancer biomarker, miRNA-155, in cell lines and human serum. The MoS_2_ flakes, as the channel sensing material used in this work, were prepared using the sequential solvent exchange method, and drop-casted onto the FET surface. The detection is based on direct hybridization between the immobilized probe miRNA-155 and the target miRNA-155. The prepared device showed a very high carrier mobility of 1.98 × 10^3^ cm^2^ V^−1^ s^−1^ (this number is much higher than expected mobilities of MoS_2_, but this is what the authors claimed), and a fairly low subthreshold swing of 48.10 mV/decade. The I_on_/I_off_ ratio (7.12 × 10^2^) reported in this work is small compared to other reports (mostly 10^5^–10^7^). In terms of miRNA detection, the prepared device achieved a LOD of 0.03 fM, in a dynamic range of 0.1 fM to 10 nM. As a selectivity test, the sensor system did not show any significant response to miRNA with one base mismatch. Lastly, this sensor system was proven to be successful in the determination of miRNA-155 human breast cancer biomarker in serum samples, which enhances its clinical applicability.

**Figure 3:**
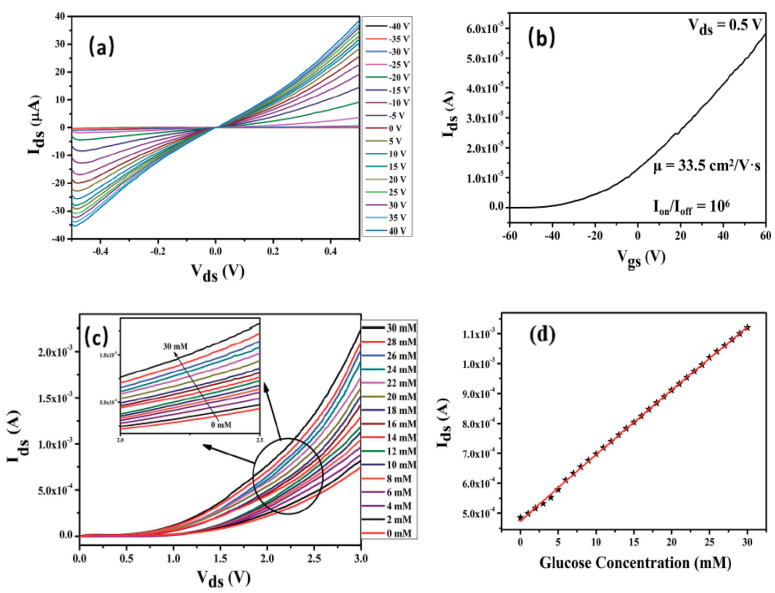
Basic electrical characterization graphs the device. (**a**) The effect of gate potential (V_g_) (from −40 to 40 V, with a step of 5 V) on device’s source-drain current (I_sd_) in the source-drain voltage (V_sd_) range of −05 to 0.5 V. (**b**) The (I_sd_-V_g_) FET characteristic curve of MoS_2_-FET showing the n-type behavior of the device. (**c**,**d**) Increase in I_sd_ with an increase in the concentration of target molecules. Reprinted with some changes from ref [86].

Shan et al. [86] reported bilayer MoS_2_-FET as a glucose biosensor, with the advantages of high stability, high sensitivity and rapid response. The electrical characteristic outputs of the introduced device were recorded in the absence of glucose. The effect of gate potential (V_g_) (from −40 to 40 V, with a step of 5 V) on the device’s source–drain current (I_sd_) in the source–drain voltage (V_sd_) range of −0.5 to 0.5 V, as shown in Figure 3a. The I_sd_ increased with the increase of positive gate potential. Furthermore, the (I_sd_-V_g_) FET characteristic curve exhibited the n-type behavior of the device with I_on_/I_off_ was found 10^6^, and the carrier mobility was found as 33.5 cm^2^ V^−1^ s^−1^, as illustrated in Figure 3b, which explains the high sensitivity of this sensor system. The presented system showed a LOD of 300 nM, and a sensitivity of 260.75 mA/mM. The current (I_sd_) was directly proportional to the glucose concentration, at constant V_g_ and V_sd_ (Figure 3c,d). This increase in current may be attributed to the n-doping of the n-type semiconductor MoS_2_ by electrons resulting from glucose oxidation. Moreover, the determination of unknown glucose concentration was achieved by a calibration curve plotted between the I_sd_ and glucose concentration. There is another interesting work presented by Lee et al. [40] about glucose biosensor using tungsten diselenide (WSe_2_) field-effect transistor biosensor (WSe_2_ BioFET) using the same concept as discussed above.

**Figure 4:**
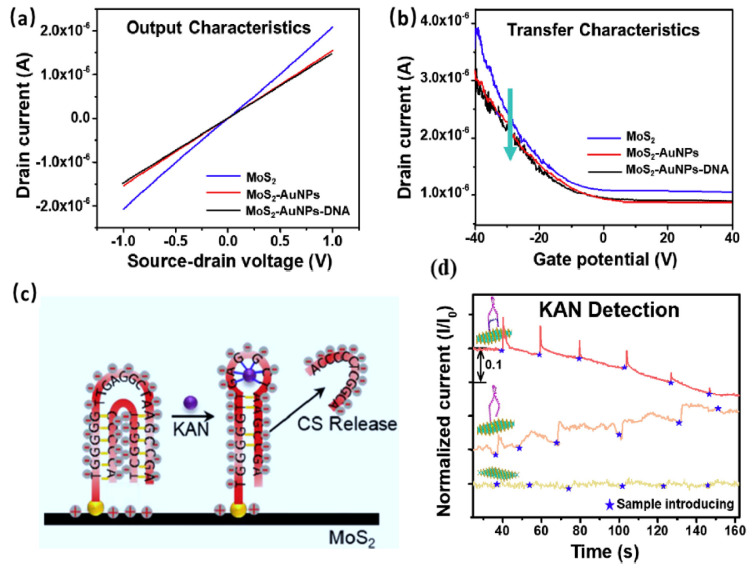
(**a**) I_sd_-V_sd_ curve showing the Ohmic contact of the device. (**b**) FET characteristic curves of the device. (**c**) The proposed mechanism of Kanamycin (KAN) replacing CS. (**d**) The sensor system, with control experiments, response to KAN at different concentrations. Reprinted from ref [41], Copyright (2019), with permission from Elsevier.

As an example of TMDC-FET biosensors for the detection of antibiotics, Chen et al. [41] developed an aptamer-MoS_2_-FET biosensor for the detection of Kanamycin (KAN). Aptamer (APT) application as a selective biorecognition element for antibiotics is promising, however, its selectivity for antibiotics is still challenging due to the wide folding of APTs and the structural similarities among antibiotics. The authors implemented MoS_2_ as the sensing material, and APT and a complementary strand DNA (CS) as recognition elements of KAN. This structure (CS-APT) of recognition elements in the proposed CS-APT-MoS_2_-FET biosensor improved the selectivity and reliability of this sensor system and reduced the device-to-device variations. Gold nanoparticles (AuNPs) were used as a linker of DNA. The device showed an Ohmic contact and p-type behavior, as shown in Figure 4a,b. MoS_2_ is expected to be n-type; however, in this work and others, it is p-type due to oxygen incorporation in the synthesis and fabrication processes [87]. The sensing mechanism is based on a replacement reaction, in which KAN binds to the APT and displaces the CS from the CS/APT/MoS_2_-FET system (Figure 4c). Two control experiments of MoS_2_-FET, and APT/MoS_2_-FET, were used in this study for a better understanding of the sensing mechanism. In the case of MoS_2_-FET, there was no change in device current with the addition of KAN. In the case of APT/MoS_2_-FET, the addition of KAN resulted in a direct increase in current due to the formation of more centered structure with higher concentration of negative charges that stimulate the positive charges of the p-type MoS_2_ and increasing current. In the main experiment, using CS/APT/MoS_2_-FET, the addition of KAN resulted in a slow current decrease, due to the time needed for KAN to displace CS, and the mechanism is hard to determine, but the authors attribute it to the decrease of negative charges in general. Figure 4d presents the results of KAN detection. The LOD, which was relatively time-dependent, was 1.06–0.66 nM, with high selectivity of KAN (selectivity coefficient of 12.8) over the other antibiotics such as amoxicillin, tobramycin, streptomycin, and chloramphenicol.

### 3.2. Black Phosphorus/Phosphorene

#### 3.2.1. Structure, Synthesis and Properties

**Figure 5:**
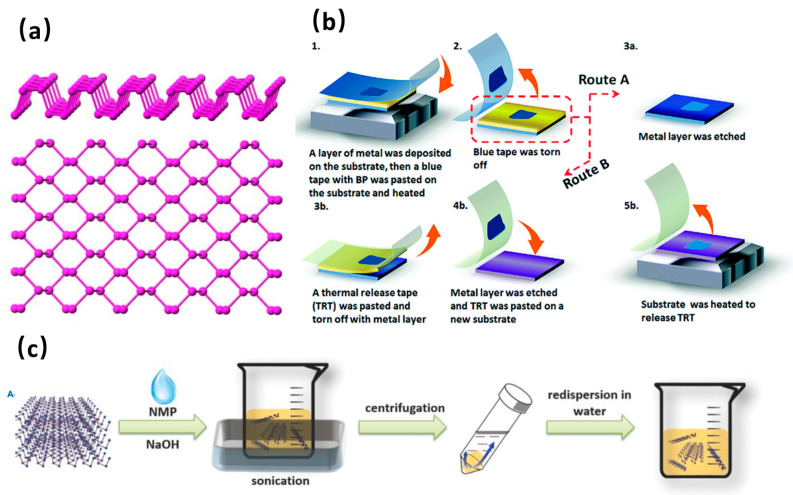
(**a**) Structure of phosphorene (side and top view) [91]. (**b**) Schematic diagram of the metal-assisted exfoliation process for few-layer black phosphorus [92]. Reprinted with permission from ref [92]. Copyright 2018, The Royal Society of Chemistry. (**c**) Schematic diagram of liquid-phase exfoliation process (basic-N-methyl-2-pyrrolidone(NMP)-exfoliated) phosphorene [93]. Reprinted with permission from ref [93]. Copyright 2015, WILEY-VCH Verlag GmbH and Co. KGaA, Weinheim.

Black phosphorus is a layered 2D Van der Waals material. It is the most stable allotropic substance among the phosphorus family. Its isolated single layer is popularly known as phosphorene and has attracted tremendous attention. The structure of phosphorene is an orthorhombic lattice and phosphorus atoms are covalently bonded to form a puckered honeycomb structure (as shown in Figure 5a) [88]. The bandgap, which ranges from 0.3 eV (for bulk black phosphorus) to 2.0 eV (for mono-layer phosphorene) is direct and thickness-dependent. The charge carrier mobility is large enough with the high hole mobility up to 1000 cm^2^ V^−1^ s^−1^, which is also thickness-dependent (reported in thickness less than 10 nm) [89,90].

Mechanical and liquid-phase exfoliations are the two most common methods to exfoliate layered phosphorene from bulk phosphorus [94,95]. Mechanical exfoliation with scotch tape to peel nanoflakes off from bulk crystals is easily operated and could yield high-quality black phosphorus/phosphorene flakes with low cost, which makes it perfect for fundamental research. On the other hand, its negative aspects are also apparent, such as the size is too small for most of exfoliated phosphorene, the process is labor-intensive and time-consuming, and the productivity is extremely low [95]. A further disadvantage is that the mechanically exfoliated phosphorene experiences significant irreversible deformations under ambient conditions and inconvenient for long-term storage. With these problems in mind, researchers are struggling to find ways to improve traditional mechanical exfoliation. Guan et al. [92] introduced a metal-assisted exfoliation method to obtain large-sized phosphene. A 10 nm gold layer (or silver layer) was deposited on the substrate at first and followed with normal mechanical exfoliation (Figure 5b). Then, the metal layer was etched with a solution. Few-layer phosphorene was produced with a 50 µm lateral size. The FET electronic properties proved the high quality of the as-fabricated phosphorene, which showed a hole mobility of 68.6 cm^2^ V^−1^ s^−1^ and the I_on_/I_off_ ratio of 200,000. Moreover, poly(dimethylsiloxane) (PDMS)-based substrate and semi-spherical PDMS stamp to help rapid peeling and transfer of phosphorene nanosheets were reported [96]. In addition, Ar^+^ plasma thinning processes after regular mechanical exfoliation to obtain controllable and homogeneous monolayer phosphorene [97] were also reported accordingly.

Liquid-phase exfoliation is another common method for preparing phosphorene. Dimethylformamide (DMF), dimethyl sulfoxide (DMSO), isopropanol (IPA), N-methyl-2-pyrrolidone (NMP), and ethanol are common solvents for black phosphorus exfoliation [98,99]. With the participation of the solvent, phosphorene was prevented from air degradation and its stability for exfoliation was improved. The phosphorene produced in the liquid phase could be stored long-term and separated by centrifugation to achieve adjustable sizes. Figure 5c illustrates a basic liquid-phase exfoliation process in NMP solvent. Bulk black phosphorus was put into the solvent, followed by a four-hour ultrasonic treatment which destructed the weak interaction between the stacked sheets [93,95]. After ultrasonic treatment, the phosphorene in NMP was separated by centrifugation. Other methods, such as electrochemical exfoliation [100], chemical transport reaction [94], solvothermal method [101], are applicable for exfoliation as well. Although a lot of literature reported successfully exfoliated phosphorene, there is still a lot of work to do before the mass-production of high-quality phosphorene can be achieved.

#### 3.2.2. Black Phosphorus/Phosphorene-FET Biosensors

**Figure 6:**
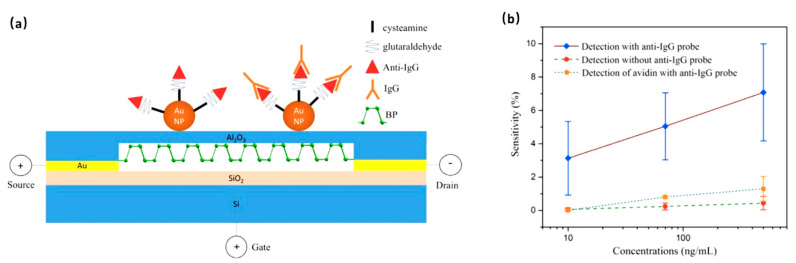
(**a**) Schematic of black phosphorus biosensor for HIgG, and (**b**) plot of sensitivity as a function of target and non-target antigen concentration [42]. Reprinted with permission from ref [42]. Copyright 2016, Elsevier B.V.

Black phosphorus and phosphene-based studies have been carried out related to biological applications, such as biomedicine and biosensing [87]. Compared with exhaustive literature on biomedicine, the work on phosphorene biosensors is far less abundant. Chen et al. [42] reported the FET biosensor with few-layer BP nanosheet serving as channel materials passivated with Al_2_O_3_ layer to detect human immunoglobulin G (HIgG). Gold nanoparticles were deposited on the surface to immobilize anti-HIgG biorecognition molecules (Figure 6). The device’s basic electrical properties showed a p-type natural of black phosphorus device. In order to test the as-fabricated sensor’s dynamic response, different concentrations of HIgG antigens from 10 ng/mL to 500 ng/mL were tested for characterization. When HIgG molecules adsorbed onto biosensor surface, the source–drain current increased with the negative gating effect added. A fast response on the order of seconds with an LOD of 10 ng/mL was reported. The sensor showed good selectivity for the target antigen compared to non-specific protein avidin (Figure 6). Kim et al. [43] successfully fabricated a few-layer black phosphorus-based biosensor to detect alpha-fetoprotein (AFP) which was called “the most reliable tumor marker for diagnosis hepatocellular carcinoma”. The surface functionalization process was conducted with poly-L-lysine linker to immobilize AFP antibodies. With the specific binding of AFP antigens and antibody, the detection of different concentrations of AFP antigen (1 ppm to 0.1 ppb) results showed a linear relationship between current and concentration with high sensitivity. Taking advantage of the fact that the phosphorus is the second most predominant mineral in the human body (1% of body weight) and the biocompatibility of biodegradation products of BP, Song et al. [44] developed a BP-FET that maintained its high mobility and on–off current ratio for ~36 h in body fluid before dissolving completely. Such a FET device has the potential to open a new way for transient biocompatible biosensors.

### 3.3. Metal Oxides

#### 3.3.1. Preparations and Properties

Metal oxides are among the most varied classes of solids. They can be classified as layered and non-layered. Examples of former include MoO_3_, WO_3_, Ga_2_O_3_ and TaO_3_, while ZnO, SnO_2_, In_2_O_3_ and CuO are examples of the latter [102]. Due to their specific structures and electrical characteristics, metal oxides have been considered as candidates to extend the library of 2D materials for transistors with a large band gap energy range (2.3–4.9 eV) and high electron mobilities (>10 cm^2^ V^−1^ s^−1^) which guarantee high sensitivity and signal-to-noise ratio in biosensing. Moreover, FETs of these materials can be processed at moderate temperatures from solutions facilitating deposition on a large scale economically and the conductivity tuned by varying crystal size, morphology, dopant, contact geometry and temperature of operation [46,102,103]. Metal oxides, to date, have been applied as electrochemical and photoelectrochemical transducers for bio/chemical sensing [30,103,104,105,106] and FET transducer for sensing of gases [107,108]. As mentioned previously, FET gas sensors of metal oxides typically operate at high temperatures, leading to higher energy needs and reliability and safety issues. On the other hand, because of oxygen atom termination of the basal surfaces, these materials are more stable in air and water [102]. As with other 2D nanomaterials, bottom-up and top-down approaches are used to synthesize many 2D metal oxide materials used in FET biosensors. The majority of the metal oxides are synthesized by hydrothermal or solvothermal procedures because these methods are facile, scalable, low temperature and low cost [109]. Typically, specific metal oxide precursors, such as metal nitrates, chlorides, and sulfates, are dissolved in water or organic solvent and reacted anywhere from 3 to 12 h or even a few days at 75–200 °C [110]. Metal oxides produced by these methods show varieties of architectures and morphologies, such as nanowires, nanowalls, nanoforests, nanoflakes, flower-like structures, and tree-like structures. [111]. Two-dimensional flakes or films may not only include layers of nanoflakes, but also aggregated nanoflakes without order. Various forms of exfoliation methods have also been implemented to achieve 2D layers of metal oxides for FET biosensors applications [45,112]. Exfoliation methods are limited to layered metal oxides.

#### 3.3.2. Metal Oxide-FET Biosensors

**Figure 7:**
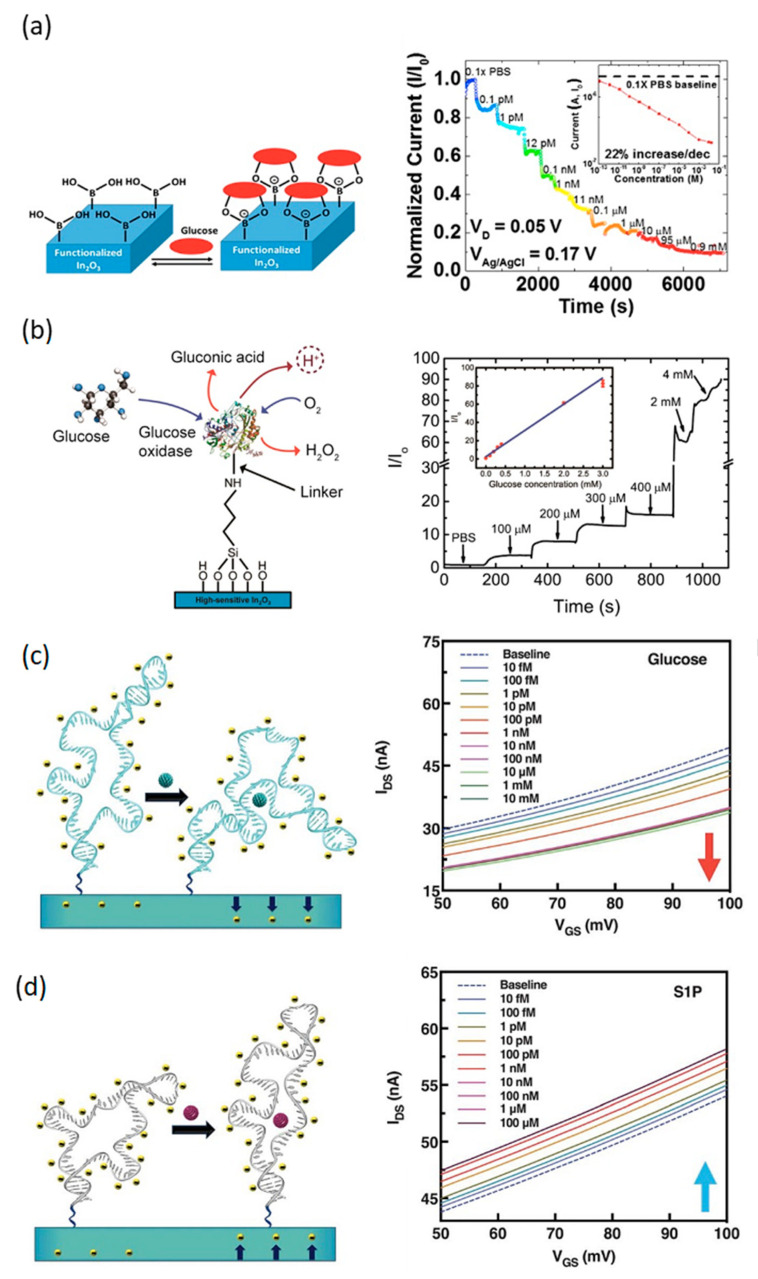
Schematic of In_2_O_3_ FET biosensors. (**a**) Illustration of the principle of glucose sensing on boronic acid-functionalized surface and responses to various concentrations of glucose [46]. Reprinted with permission from ref [46]. Copyright 2017, American Chemical Society. (**b**) Illustration of D-glucose sensing via glucose oxidase to produce gluconic acid and hydrogen peroxide (left), and responses to physiologically relevant D-glucose concentrations (right) (inset shows data from five devices) [47]. Reprinted with permission from ref [47]. Copyright 2015, American Chemical Society. Mechanism of aptamer target-induced reorientations within or near the Debye length of semiconductor channels. (**c**) Aptamers reorient closer (e.g., dopamine, glucose) to decrease transconductance (left). Transfer curves of glucose aptamer–FETs showed reductions in source-drain currents (right), (**d**) Aptamers reorient away from semiconductor channels (e.g., serotonin, S1P) to increase transconductance (left). Transfer curves of S1P aptamer–FET transfer curves increased in response to target concentrations (right). Reprinted with some changes from ref [115]. Reprinted with permission from ref [115]. Copyright 2018, Science.

Two-dimensional metal oxides have an extensive application as optical, electronic, and sensing semiconductors. Compared with a large number of one-dimensional metal oxide-based FET biosensors reported [113,114], 2D metal oxide-based FET biosensors still have a large space in FET biosensors applications. Herein, we briefly introduce several metal oxide-based FET biosensors. When considering various metal oxides, In_2_O_3_ has yielded many FET biosensors with good performance. Chen et al. [46] presented a 2D In_2_O_3_-based FET biosensor which achieved specific detection of glucose with an extremely low limit of detection (<7 fM) and showed high sensitivity. Boronic acid and glucose, respectively, acted as the receptor and target molecules (Figure 7a). The transfer curves in 0.1 M buffer solution showed clear ohmic behavior at low bias voltage and all I-V curves showed a turn-on voltage of −0.316 V with I_on_/I_off_ ratio of 10^4^. The biosensor response was linearly related to glucose concentration over a broad dynamic range from 10^−11^ to 10^−5^ M, as shown in Figure 7a. The device performances, both with respect to dynamic range and limit of detection, of this sensor was superior to other non-enzymatic FET glucose sensors using boronic acid as recognition molecule in conjunction with carbon nanotubes, graphene or reduced graphene oxide as semiconductor channel.

The Tseng group from the University of California, Los Angeles (UCLA) [47] demonstrated an In_2_O_3_-based FET biosensor with ultrathin polyimide (PI) film as a substrate for conformal bioelectronics. The enzymatic oxidation of D-glucose with glucose oxidase was applied to specifically detect only D-glucose not L-glucose with a low driving voltage. The mobility and and I_on_/I_off_ ratio of the In_2_O_3_-based FET biosensor were 20 cm^2^ V^−1^ s^−1^ and larger than 10^7^, respectively. The sensor detected physiologically relevant D-glucose concentrations (Figure 7b). The mechanism of glucose sensing was based on the protonation of In_2_O_3_ surface by gluconic acid produced during glucose oxidase catalyzed oxidation of D-glucose. The reported biosensor has the potential for applications in wearable, non-invasive health-monitoring technologies such as glucose levels in tears.

Highly sensitive In_2_O_3_-based FET biosensors with DNA aptamers for the detection of small electroneutral molecules, such as dopamine, glucose, serotonin, and sphingosine-1-phosphate (S1P) in undiluted physiological fluids of high-ionic strength was reported by the Weiss group from UCLA [115]. The DNA aptamers for the target compounds were selected by solution-phase systematic evolution of ligands by exponential enrichment (SELEX) and immobilized on the semiconductor In_2_O_3_ sensing channel. The sensing mechanism was the modulation in the gate conductance of the device as a result of a target-induced change in the confirmation of negatively charged phosphodiester backbones of immobilized aptamers. For example, when the glucose aptamer-FET was exposed to glucose, charged backbones of aptamers moved closer to the semiconductor channel causing an increase in electrostatic repulsion and a decrease of device transconductance (Figure 7c). On the other hand, S1P aptamers moved away from the channel surface when target S1P was captured by the aptamers, resulting in an increase of the device transconductance (Figure 7d). The integration of highly sensitive In_2_O_3_-based FET and specific stem-loop receptor overcame the limitations of traditional FET biosensors in detecting molecules/targets in high ionic strength solutions because of shielding created by the electrical double layer, i.e., Debye length and/or small molecules with no or few charges.

Balendhran et al. [45] reported a liquid exfoliation method to obtain 2D α-MoO_3_ nanoflakes with lateral dimensions in the range 50–150 nm. Then, a MoO_3_ electron conduction channel was drop-casted on a rough alumina substrate to obtain a FET biosensor for bovine serum albumin detections. The platform showed a fast response time of less than 10 s and LOD of 250 μg/mL. As more attention has been paid in 2D metal oxide materials, they will provide great opportunities in the detection of biomolecule applications.

### 3.4. h-BN

Hexagonal boron nitride has a similar chemical structure to graphene and has a stoichiometry of 1:1 of B and N. With the help of the covalent B-N bond, the localized electronic state provides h-BN with a high band gap from 5 eV to 6 eV and thereby excellent electrical insulation properties [116]. Like most 2D materials, single- or few-layer h-BN can be produced by either exfoliating from bulk crystals of boron nitride or by the CVD method [117]. The excellent electrical insulation property makes 2D h-BN a perfect alternative dielectric for layered 2D material based heterostructures and has recently exhibited considerable improvements on channel mobility [118,119]. Dean et al. [120] fabricated and characterized graphene on h-BN substrates devices. Nanosheets h-BN were produced from bulk single-crystal h-BN by exfoliation. Its one-atom structure contributed to the smooth surface and reduced roughness compared to SiO_2_. Then, graphene was transferred by a typical polymethylmethacrylate (PMMA)-based method to build graphene/h-BN heterostructures. Electronic transport measurement results showed super excellent hall mobility of monolayer graphene device (140,000 cm^2^ V^−1^ s^−1^) which could compare with SiO_2_ supported devices. In another report by Joo et al. [121], the heterostructure device of 2D MoS_2_ on h-BN substrate was found to have much higher n-doping effect compared to MoS_2_ directly on SiO_2_/Si substrate. This was attributed to the h-BN layer helping reduce the oxygen p-doping with SiO_2_, thereby lowering Schottky barrier height in MoS_2_/hBN heterostructure. Similarly, h-BN/MoS_2_/h-BN heterostructure was reported by Saito et al. [122] as well. When the conductance characterization was conducted within the linear region, hysteresis was small enough to be negligible. Meanwhile, in the nonlinear, hysteresis behaviors could be detected under dark condition. They also reported the illumination effects on the hysteric behaviors. When conductance characteristics operated under illumination, hysteric behaviors in the nonlinear region disappeared. The above results, while they do not present specific examples of applications of h-BN-based biosensors, provide illustrations of potentials of heterostructures of h-BN with other 2D materials in construction of biosensors with ultrahigh sensitivity.

## 4. Summary, Challenges and Future Perspectives

FET biosensors are very promising for clinical applications as early diagnostic tools, due to their high sensitivity, rapid response, low power operation, label-free working environment, and feasibility for commercialization. The semiconducting channel of nanomaterials plays a crucial rule in the sensing process, and alongside the recognition element, they determine the sensing characteristics of a FET biosensor. Graphene has shown to be successful as a channel material due to its 2D layered structure, high surface area, and very high charge carrier mobility. However, this success is limited, as graphene has no intrinsic band gap, i.e., semimetallic, which lowers its current switching ratio (I_on_/I_off_ ratio is 1 to 2), and, in role, lowers the sensitivity of its corresponding graphene-FET biosensors. Other materials such as SiNWs and CNTs were reported as promising FET sensing channel materials in a huge number of studies, however, they suffer from difficulty in achieving high reproducibility, as discussed in the introduction of this article.

**Figure 8:**
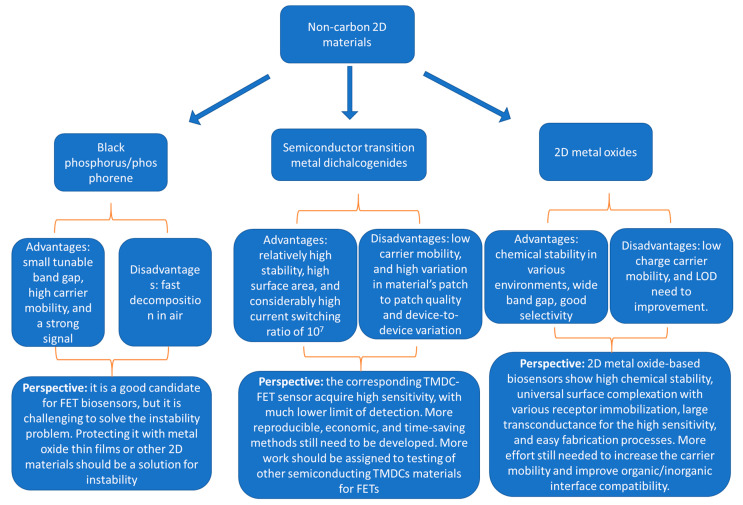
A schematic diagram summarizing the non-carbon 2D materials, their advantages and disadvantages as channel materials for FET biosensor applications, and the authors perspectives about them.

Keeping all that in mind, non-carbon 2D materials, which include TMDCs, BP, 2D metal oxides, h-BN, and others, represent the new candidates. Figure 8 is a schematic diagram summarizing the different types of non-carbon 2D materials used in FET biosensors and highlighting their advantages, disadvantages and future perspectives. Semiconducting TMDCs are the most promising among them, due to their 2D layered structure, relatively high stability, high surface area, and considerably high current switching ratio of 10^3^–10^7^ that increases the corresponding TMDC-FET sensor sensitivity and enables much lower limit of detection. Mono-to-few layer BP is another new promising material for FET biosensors, as it has a higher charge carrier mobility compared to TMDCs, and a higher conductivity with a lower band gap. BP has a lower Schottky barrier due to its better wavefunction matching with metal contacts. Non-carbon-FET biosensors for the detection of nucleic acids, proteins, cancer biomarkers, glucose, and others, have been successfully conducted on a lab scale. However, much effort is still required to reach clinical applications. The main challenges are in achieving the stability and reproducibility of these 2D-FETs devices, as well as the materials and devices fabrication methods. CVD is a promising method for the synthesis of TMDCs, but it lacks the high reproducibility from a batch to another and results in device-to-device variation. The variation can be due to changes in grain size, level of defects, film continuity, and more. Moreover, transferring the CVD-synthesized materials to the desired substrate, to build the FET sensor, introduces many defects that negatively affect the device performance. Chemical and liquid phase exfoliation methods are promising in terms of scaling up, but the drop-casting of the materials on the surface of FET is not that highly reproducible either. BP has another problem, which is its very low stability, as it degrades in air and moisture even in a few hours. In addition, biosensor applications make materials more susceptible to degradation, as they involve liquids in contact with these materials for a long time. Two-dimensional metal oxides-based biosensors are very promising due to their high chemical stability, and universal surface complexation with various receptors. In addition, metal oxides are easy to synthesize, process, and load on substrates. However, more efforts are still needed to increase carrier mobility and improve organic/inorganic interface compatibility. Monolayer h-BN is an excellent alternative for dielectric layers (e.g., SiO_2_) in FET, where it helps reduce the oxygen p-doping of SiO_2_, thereby lowering Schottky barrier height, and reduces surface roughness scattering and hence it improves the carrier mobility of the top sensing material such as graphene or TMDCs.

Another problem with non-carbon 2D materials can be that these materials are still new, and their fabrications routes are not robust and clear enough to a large portion of the research community. It is worth mentioning that the number of studies conducted on TMDCs-, BP-, or 2D metals oxides-FET biosensors is significantly limited over the last five years. Even though, the presented limited number of studies is mainly focused on MoS_2_-FETs, while the other semiconducting TMDCs, such as MoSe_2_, WS_2_, and WSe_2_, were just not getting their chances yet. To overcome these problems and open the way for their FET biosensors, more reproducible, economic, and time-saving methods still need to be developed. A complete library of the non-carbon 2D materials, based on matching their electronic and semiconducting properties and tabulating them for an easier choice for FETs and commercialization, is still needed. More work should be assigned to the testing of other semiconducting TMDC materials for FETs.

## Figures and Tables

**Table 1:** Overview of literature reports on non-carbon 2D material-based FET biosensors.

2D Material	2D Thickness [nm]	Mobilities [cm^2^ V^−1^ s^−1^]	I_on_/I_off_ Ratio	Target Molecule	Detection Limit/Range	Response Time	Reference
MoS_2_	-	1.98 × 10^3^	7.12 × 10^2^	miRNA-155	0.03 fM	40 min	[39]
WSe_2_	-	-	>10^5^	Glucose	1.0–10 mM	-	[40]
MoS_2_	0.7	-	3.6–3.8	Kanamycin	1.06–0.66 nM	20 min	[41]
BP	10–60	-	-	IgG	10 to 500 ng/mL	on the order of seconds	[42]
Phosphorene	-	-	-	Alpha-fetoprotein	0.1 ppb–1 ppm	-	[43]
BP	30–50	468	1200	-	-	-	[44]
MoO_3_	1.4–2.8	1100	-	Bovine serum albumin	250 μg/mL–25 mg/mL	<10 s	[45]
In_2_O_3_	4	19	-	Glucose	10^−11^–10^−5^ M	-	[46]
In_2_O_3_	3.5	20	>10^7^	Glucose	0.1–0.6 mM	-	[47]
